# Pandemic (H1N1) 2009 Virus in Swine Herds, People’s Republic of China

**DOI:** 10.3201/eid1709.101916

**Published:** 2011-09

**Authors:** Hongbo Zhou, Can Wang, Ying Yang, Xuebo Guo, Chao Kang, Huanchun Chen, Meilin Jin

**Affiliations:** Author affiliation: State Key Laboratory of Agricultural Microbiology of the Huazhong Agricultural University, Wuhan, People’s Republic of China

**Keywords:** virus diseases, RNA virus infections, influenza, viruses, pandemic (H1N1) 2009 virus, pigs, People’s Republic of China, letter, Suggested citation for this article: Zhou H, Wang C, Yang Y, Guo X, Kang C, Chen H, et al. Pandemic (H1N1) 2009 in swine herds, People’s Republic of China [letter]. Emerg Infect Dis [serial on the Internet]. 2011 Sep [date cited]. http://dx.doi.org/10.3201/eid1709.101916

**To the Editor:** During March and early April 2009, a new swine-origin influenza A (H1N1) virus emerged in Mexico and the United States; this virus subsequently spread across the globe by human-to-human transmission at an unprecedented rate. Pandemic (H1N1) 2009 virus also affected pigs. On May 2, 2009, the Canadian Food Inspection Agency notified the World Organisation for Animal Health that the novel influenza A virus had been confirmed on a pig farm in Alberta, Canada. Infection of pigs with pandemic (H1N1) 2009 virus has been observed in multiple countries (*1*). In this study, we report transmission of pandemic (H1N1) 2009 virus from humans to pigs in the People’s Republic of China.

During August 2009–April 2010, swine influenza virus surveillance was conducted in the provinces of central and eastern China, including Henan, Hubei, Hunan, Jiangxi, and Anhui. A total of 1,021 samples, comprising tracheal mucus swabs and lungs, from pigs on 30 farms, were collected, and dozens of swine influenza viruses, H1N1, H1N2, H3N8, H9, and H10 subtypes, were isolated. Eight isolates were subtype H1N1, including 4 novel pandemic (H1N1) 2009 viruses: A/swine/Nanchang/3/2010 (H1N1) (GenBank accession nos. JF275917–24), A/swine/Nanchang/5/2010 (H1N1) (GenBank accession nos. JF275933–40) and A/swine/Nanchang/6/2010 (H1N1) (GenBank accession nos. JF275941–48), which were isolated from tracheal mucus, and A/swine/Nanchang/F9/2010 (H1N1) (GenBank accession nos. JF275925–32), isolated from the lung. Pigs from which the novel viruses were isolated showed mild respiratory signs, including depression, cough, and transient increase in body temperature. Compared with the sequence of A/California/04/2009 (H1N1), genomic sequencing of the 4 pandemic viruses showed 15 common point mutations, such as polymerase basic protein 2, T588I; polymerase acidic protein, A70V, P224S, D547E; hemagglutinin, P100S, D103E, S145P, T214A, S220T, I338V; nucleocapsid protein, V100I, H289Y; neuraminidase, V106I, N248D; and nonstructural protein, I123V.

Recent studies have shown that the novel pandemic (H1N1) 2009 human influenza viruses were almost avirulent for mice (50% mouse lethal dose >10^6^ PFU for A/CA/04/09 [*2*,*3*]). In this study, mice were anesthetized with ketamine/xylazine, as described (*4*) and 50 μL of phosphate-buffered saline containing the indicated doses (10^5^ 50% egg infectious dose) of the 4 viruses were instilled into anesthetized mice through nostrils. Interestingly, the 4 pandemic (H1N1) 2009 viruses isolated from pigs could cause systemic infection on mice. All mice had extensive loss of body weight, and some of the infected mice died within 2 weeks postinfection (data not shown).

To detect whether the 4 isolated pandemic (H1N1) 2009 viruses could cause clinical diseases in pigs and what kinds of pathologic damage could be induced in infected pigs, pigs were intratracheally challenged with the 4 pandemic (H1N1) 2009 viruses at a dose of 10^7^ 50% egg infectious dose and monitored for clinical signs and pathologic changes. Clinical signs in pigs were mild, and no deaths occurred. Except for slightly labored breathing, no infected pigs showed dominant signs, such as cough, nasal discharge, facial edema, and dyspnea. Body temperatures of infected pigs started to increase at 2 days postinfection (dpi), peaked ≈3 dpi at 41.5°C, and returned to the initial temperature by 5 dpi.

We observed necrosis of the tracheal wall and pneumonia foci in the 4 pandemic (H1N1) 2009 virus–infected pigs. Hematoxylin and eosin–stained sections of the trachea from the infected pigs showed mild necrotizing tracheitis. The necrotic epithelial cells were present in the lumen, and a mixed inflammatory cell infiltrate was present throughout (Figure). In the lung, we also observed alveolar septal edema and interstitial inflammatory cell infiltrates, as well as histologic changes, including alveolar epithelial hyperplasia, a mixed inflammatory infiltrate, and interstitium broadening (Figure). Additionally, immunohistochemical analysis for the distribution of viral antigens showed positive staining in bronchial epithelial cells and alveolar pneumocytes.

**Figure:**
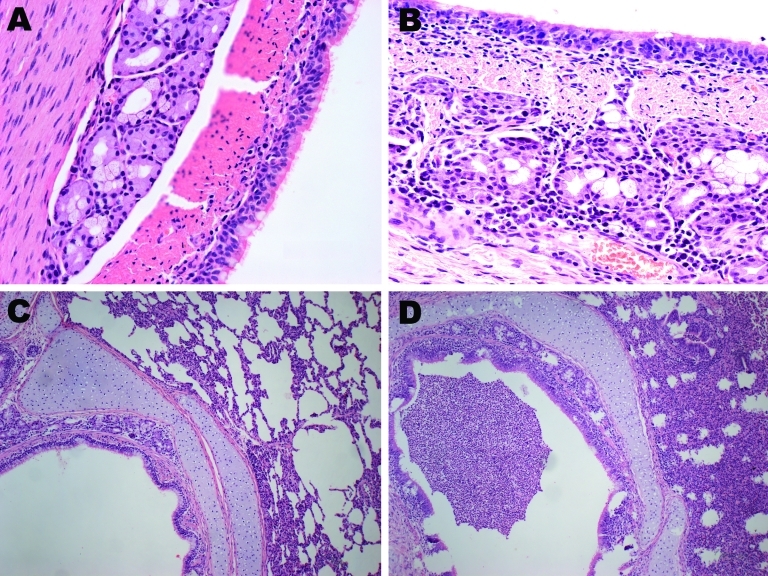
Hematoxylin and eosin–stained trachea and lung controls and samples from pigs infected with pandemic (H1N1) 2009 virus. A) Control trachea sample; B) mixed inflammatory cell infiltrate present throughout trachea sample from infected pig. C) Control lung sample; D) mixed inflammatory infiltrate and interstitium broadening in lung sample from infected pig. Original magnifications: panels A and B, ×40; panels C and D, ×10.

Previous studies showed that pandemic (H1N1) 2009 may have become established in swine populations in Canada, Norway, and Hong Kong (*1**,**5**–**8*). The human-to-pig transmission of pandemic (H1N1) 2009 may substantially affect virus evolution and subsequent epidemiology. Although the pandemic was mild, the virus could develop further reassortment in swine and gain virulence. On the other hand, subtype H5N1 and H9N2 viruses have become established in pigs, so the introduction of pandemic (H1N1) 2009 virus to pigs has provided the possibility for the incorporation of avian virus genes into mammalian-adapted viruses. That transmission could occur from humans to pigs and vice versa is especially troublesome. Given the possible production of novel viruses of potential threat to public health, we should emphasize influenza surveillance in pigs and establishment of the genetic basis of the viral genome for rapidly identifying such reassortment events.
